# Impact of a change of bronchodilator medications in a hospital drug formulary on intra- and out-of-hospital drug prescriptions: interrupted time series design with comparison group

**DOI:** 10.1186/s13012-020-00996-y

**Published:** 2020-05-14

**Authors:** Raquel Vázquez-Mourelle, Eduardo Carracedo-Martínez, Adolfo Figueiras

**Affiliations:** 1 Galician Health Service (Servicio Gallego de Salud - SERGAS), Galicia Regional Authority, Administrativo San Lázaro s/n, 15703 Santiago de Compostela, Galicia Spain; 2 Santiago de Compostela Health Area Authority, Galician Health Service, Rúa da Choupana, s/n, 15706 Santiago de Compostela, A Coruña, Galicia Spain; 3 Department of Preventive Medicine and Public Health, Faculty of Medicine, Consortium for Biomedical Research in Epidemiology & Public Health (CIBER en Epidemiología y Salud Pública - CIBERESP), and Health Research Institute of Santiago de Compostela (Instituto de Investigación Sanitaria de Santiago de Compostela - IDIS), University of Santiago de Compostela, Praza Seminario de Estudos Galegos, s/n, 15705 Santiago de Compostela, Galicia Spain

**Keywords:** Bronchodilator agents, Hospital formulary, Drug prescription, Interrupted time series studies, Pharmacoepidemiology

## Abstract

**Background:**

Hospital drug formularies are reduced lists of drugs designed to optimise inpatient care. Adherence to the drugs included in such formularies is not always 100% but is generally very high. Little research has targeted the impact of a change in these formularies on outpatient drug prescriptions. This study therefore sought to evaluate the impact of a change affecting bronchodilator medications in a hospital drug formulary on intra- and out-of-hospital drug prescriptions in a region in north-western Spain. Two new drugs belonging to this same class were brought onto the out-of-hospital market, overlapping with the intervention.

**Methods:**

We used a natural before-after quasi-experimental design with control group based on monthly data. The intervention evaluated was the modification of a hospital drug formulary, which involved withdrawing salmeterol/fluticasone in order to retain formoterol/budesonide as the sole inhaled corticosteroid and long-acting beta-agonist (ICS/LABA). Using official data sources, we extracted the following dependent variables: defined daily doses (DDD) per 1000 inhabitants per day, DDD per 100 bed-days, and cost per DDD.

**Results:**

Intra-hospital use showed a 173.2% rise (95% CI 47.3–299.0%) in the medication retained in the formulary, formoterol/budesonide, and a 94.9% drop (95% CI 77.9–111.9%) in the medication withdrawn from the formulary, salmeterol/fluticasone. This intervention led to an immediate reduction of 75.9% (95% CI 82.8–68.9%) in the intra-hospital cost per DDD of ICS/LABA. No significant changes were observed in out-of-hospital use.

**Conclusions:**

Although this intervention was cost-effective in the intra-hospital setting, the out-of-hospital impact of a change in the drug formulary cannot be generalised to all types of medications and situations.

Contributions to the literature

- It appears that the impact induced by a change in hospital pharmacotherapy guidelines on intra- and out-of-hospital drug prescriptions cannot be generalised to all compounds and circumstances.
- Beyond the change in hospital pharmacotherapy guidelines, there are external factors, such as the recent appearance on the market of new medications in the same group, which could serve to neutralise the possible effect of a guideline change at an out-of-hospital level.
- From a methodological standpoint, the availability of a control group in interrupted time series studies is of great relevance.

## Introduction

Pharmaceutical products account for a substantial proportion of total health-care expenditure in developed countries [1]. The growth of this expense is attributable to various factors, such as the appearance of new drugs [2], changes in clinical practice [1], and/or hospital-induced drug prescribing among primary care physicians [2–5], and differs between countries and drug therapeutic classes [1].

In Spain, the proportion of out-of-hospital pharmaceutical cost to total health-care cost is around 16–18% [5, 6], twofold that of countries such as Denmark (6.8%) or Sweden (9.9%) [6]. In order to improve drug prescribing, different strategies (educational, administrative, management, regulatory, etc.) have been implemented [6, 7] but have not always attained the desired goals.

A traditional tool of rational use in the hospital setting is the hospital drug formulary (HDF) or reduced list of drugs [8–13] for use on hospitalised patients. However, there is very little evidence of the impact of a change in the HDF on the intra- and out-of-hospital setting [14, 15, 16, 17]. Recent studies conducted by our group found that a change in low molecular weight heparins (LMWH) in the HDF also had an important impact (induction of prescription) on the out-of-hospital setting [17]. Even so, it is not known whether this effect is generalisable or, on the contrary, depends on other factors, such as therapeutic group, type of formulary change (withdrawal, restriction on use, or inclusion), or other contextual factors, e.g. the marketing of new drugs. Taking the theoretical model into account, we postulated the following hypothesis, namely, that a change in the HDF would influence intra- and out-of-hospital prescribing patterns, not only in terms of the target medication, but also in terms of the other medications belonging to the same therapeutic group.

Accordingly, this study set out to evaluate the impact on these two health-care settings (hospital and/or primary care) of a change in a hospital drug formulary affecting inhaled fixed-dose corticosteroids and long-acting beta2-agonists (ICS/LABA), a therapeutic class in which two new drugs were brought onto the out-of-hospital market, overlapping with the intervention.

## Material and methods

### Setting

The study was undertaken in Galicia, a region in north-west Spain with a population of 2,708,339 in 2017. A total of 98.2% of the population is covered by the Public Health Service under a public health insurance system. The Galicia Public Health Service has a daily average of 38,050 health professionals, 2601 of whom are primary care physicians. The region is divided into 7 health areas. Health-care services are free of charge, and while intra-hospital drug provision is likewise free of charge, out-of-hospital drug provision is subject to a monetary contribution (co-payment).

The official prices of subsidised drugs dispensed at retail pharmacies are set by the state. When it comes to intra-hospital use, however, individual hospitals are free to negotiate their prices with pharmaceutical companies. Competence to decide on a drug’s inclusion in or withdrawal from a given hospital’s drug formulary lies with the collegiate body known as the Intra-hospital Pharmacy and Therapeutics Committee.

Respiratory tract diseases account for 11.6% of all hospital discharges and are the fourth leading cause of hospitalisations in Spain [18]. In 2015, the out-of-hospital costs per defined daily doses (DDD) of the respective ICS/LABA were as follows: formoterol/budesonide, €1.14; formoterol/fluticasone, €1.32; vilanterol/fluticasone, €1.71; formoterol/beclometasone, €1.74; and salmeterol/fluticasone, €2.47. Formoterol/fluticasone and vilanterol/fluticasone are combinations which were brought onto the market shortly before the intervention evaluated by this study.

### Study design

We used a quasi-experimental ecological design with a control group, based on monthly drug-use data from January 2014 to January 2018*.* The longitudinal nature of the data and the fact of having a control group lent the design a special robustness [19], owing to the fact that in these types of designs, confounding bias is controlled for by design, with each study unit (study population area) being compared in the post-intervention versus the pre-intervention period. Only time-dependent confounding variables might affect the study, and a control group was thus used to control for these [20, 21].

This study was neither sponsored nor funded by any company. The methodological and reporting guidelines recommended by Jandoc et al. for interrupted time series studies were followed to ensure standardisation and enhance the quality of the reporting [22].

### Intervention

We studied a change in the hospital drug formulary affecting the ICS/LABA therapeutic group of pulmonary inhalation therapy drugs. The change consisted of withdrawing salmeterol/fluticasone in order to retain just one ICS/LABA, namely, formoterol/budesonide. This change was made in January 2015.

### Intervention group

In 2016, the health area targeted by the intervention had a catchment population of 447,699, a 1049-bed referral hospital, 75 primary care centres, and a daily average of 1710 physicians (active in primary and hospital care, including emergency units or services). Table 1 shows the population distribution of the intervention and control groups.

**Table 1:** Intervention and control groups: demographic data

Characteristics	Intervention group	Control group
	*N* = 445,474	*N* = 549,292
Age—*n* (%)		
0–18 years	67,347 (15.1)	83,157 (15.1)
19–30 years	47,669 (10.7)	56,328 (10.2)
31–50 years	134,660 (30.3)	171,673 (31.2)
51–70 years	119,066 (26.7)	144,879 (26.4)
> 70 years	76,732 (17.2)	93,255 (17.0)
Sex—*n* (%)		
Men	215,653 (48.4)	263,249 (47.9)
Women	229,821 (51.5)	286,043 (52.1)

### Control group

Another health area of similar population characteristics and health-care resources, coming under the same public health service, served as the control health area (Table 1). The proposed study intervention was not implemented here, with the two ICS/LABA then in use, formoterol/budesonide and salmeterol/fluticasone, thus being retained in the local formulary. In 2017, this area had a catchment population of 550,473, a 1359-bed referral hospital, 71 primary care centres, and a daily average of 1744 physicians.

### Data source

All data were sourced from official Public Health Service records, in which the registration of data is population-based (no sampling) and exhaustive (practically no risk of under-reporting), inasmuch as it is linked to pharmacy accounting and invoicing aspects. There was no change in the data registration system across the study period.

In the case of intra-hospital data, we used the Hospital Pharmacy Corporate Information System (*Sistema de Información Corporativo de Farmacia Hospitalaria*), which furnishes the figures for all drugs dispensed by Public Health Service hospital pharmacies for use on hospitalised patients.

Out-of-hospital prescriptions were obtained from the Official Pharmacy Information System for Complex Pharmacy Service Analyses (*Sistema Corporativo de Información de Análisis Complejos Prestación Farmacéutica*). This shows all medications dispensed by community pharmacies in the health area on the basis of official medical prescriptions billed to the Public Health Service.

### Definition of variables

We calculated the number of monthly DDD [23] of the five ICS/LABA marketed, namely, formoterol/budesonide, formoterol/beclometasone, vilanterol/fluticasone, formoterol/fluticasone, and salmeterol/fluticasone.

The DDD is the assumed average maintenance dose per day for a drug used for its main indication in adults [24]. We calculated the DDD per 1000 inhabitants per day (DDD/TID), and the DDD per 100 bed-days (DDD/100 b-d) [25] across the study period.

These are the basic units of consumption used internationally to examine drug use at a population or hospital level and are not affected by the different forms of drug presentation [23].

We took into account the mean population of the study areas to calculate DDD/TID [24] and mean hospital stays to calculate DDD/100 b-d [24]. For both DDD/TID and DDD/100 b-d, we calculated the monthly values across the study period.

Cost per DDD was calculated by dividing the total monthly cost by the number of DDD prescribed that month.

### Statistical analysis

The model selected uses aggregate data collected over equally spaced intervals (monthly).

The number of measurements obtained was 13 points pre-intervention and 35 points post-intervention (Table S2), in the period from January 2014 to January 2018. For statistical analysis purposes, we used interrupted time series (ITS) analysis and constructed a segmented regression model with a control group [26]. This is a method increasingly used in drug-use analysis [22, 27, 28]; its principal strength lies in the fact that it evaluates the effect of the intervention by explaining all the important trends which precede the intervention [19]. This model allows one to evaluate the longitudinal effect of interventions where randomisation is not feasible, and one has the monthly data sequence of a large historical series [22]*.* Furthermore, if adjustment is made for the values of the control area, this will highlight the effects of the intervention, by eliminating the possible influence of external co-interventions, such as alerts, commercial promotions, or the marketing of new drugs.

It should be noted that the inclusion of the terms ZX, ZT, and XT, as well as X, T, and Z, responds to the need to maintain the hierarchical principle and to the need to maintain them even though they may not be statistically significant.

To do so, we used the following equation:

$$ \mathrm{Yt}={\upbeta}_0+{\upbeta}_1{\mathrm{T}}_{\mathrm{t}}+{\upbeta}_2\mathrm{Xt}+{\upbeta}_3{\mathrm{X}}_{\mathrm{t}}{\mathrm{T}}_{\mathrm{t}}+{\upbeta}_4\mathrm{Z}+{\upbeta}_5{\mathrm{ZT}}_{\mathrm{t}}+{\upbeta}_6\mathrm{ZXt}+{\upbeta}_7{\mathrm{ZX}}_{\mathrm{t}}{\mathrm{T}}_{\mathrm{t}}+\mathrm{et} $$

where:

- Yt is the dependent variable with monthly values (DDD/TID, DDD/100 b-d, and cost per DDD).
- β0 represents the initial level of the dependent variable.
- β1 is the slope of the dependent variable until the implementation of the intervention.
- Tt is the number of months from study onset.
- β2 represents the change in the level of the dependent variable which occurs in the immediate post- versus the immediate pre-intervention period.
- Xt is a dummy variable representing the intervention (pre-intervention period, 1; post-intervention period, 0).
- β3 represents the difference between the pre- and post-intervention period in the slope of the dependent variable.
- XtTt is an interaction term.
- In the pre-intervention period, β4 represents the difference in level of the dependent variable between the intervention and control areas.
- *Z* is a dummy variable that denotes cohort allocation (intervention = 0, control = 1).
- In the pre-intervention period, β5 represents the difference in slope of the dependent variable between the intervention and control areas.
- ZTt is an interaction term.
- β6 indicates the difference between the control and intervention groups in terms of the level of the dependent variable immediately after the intervention.
- ZXt is an interaction term.
- β7 represents the difference between the control and intervention groups in terms of the difference between the slope of the dependent variable pre- and post-intervention.
- ZXtTt is an interaction term.
- et is the random error term.

To evaluate possible autocorrelation, we introduced auto-regressive terms into the model.

Based on the regression coefficient β6, we calculated the percentage reduction in each ICS/LABA with respect to the situation immediately preceding the change in the hospital drug formulary.

Two sensitivity analyses were performed: the first to assess the influence of the control group on the outcomes, applying a classic ITS model (without control group; Table S1), and the second to analyse the other respiratory system medications with indication for asthma or COPD, in order to ascertain whether there was some type of impact on these.

The statistical software programme used for analysis purposes was IBM SPSS Statistics.

## Results

Table 1 describes the characteristics of the population of the study areas by sex and age. As can be seen, both were very similar in terms of age and sex structure. Table S2 shows the mean (SD) DDD/100 b-d for ICS/LABA and the cost (€/DDD) pre- and post-intervention in the study and control groups in intra-hospital settings, and it also shows the mean (SD) DDD/TID for the sum of ICS/LABA and the cost (€/DDD) in the study and control groups in out-of-hospital settings.

### Intra-hospital use

**Fig. 1:**
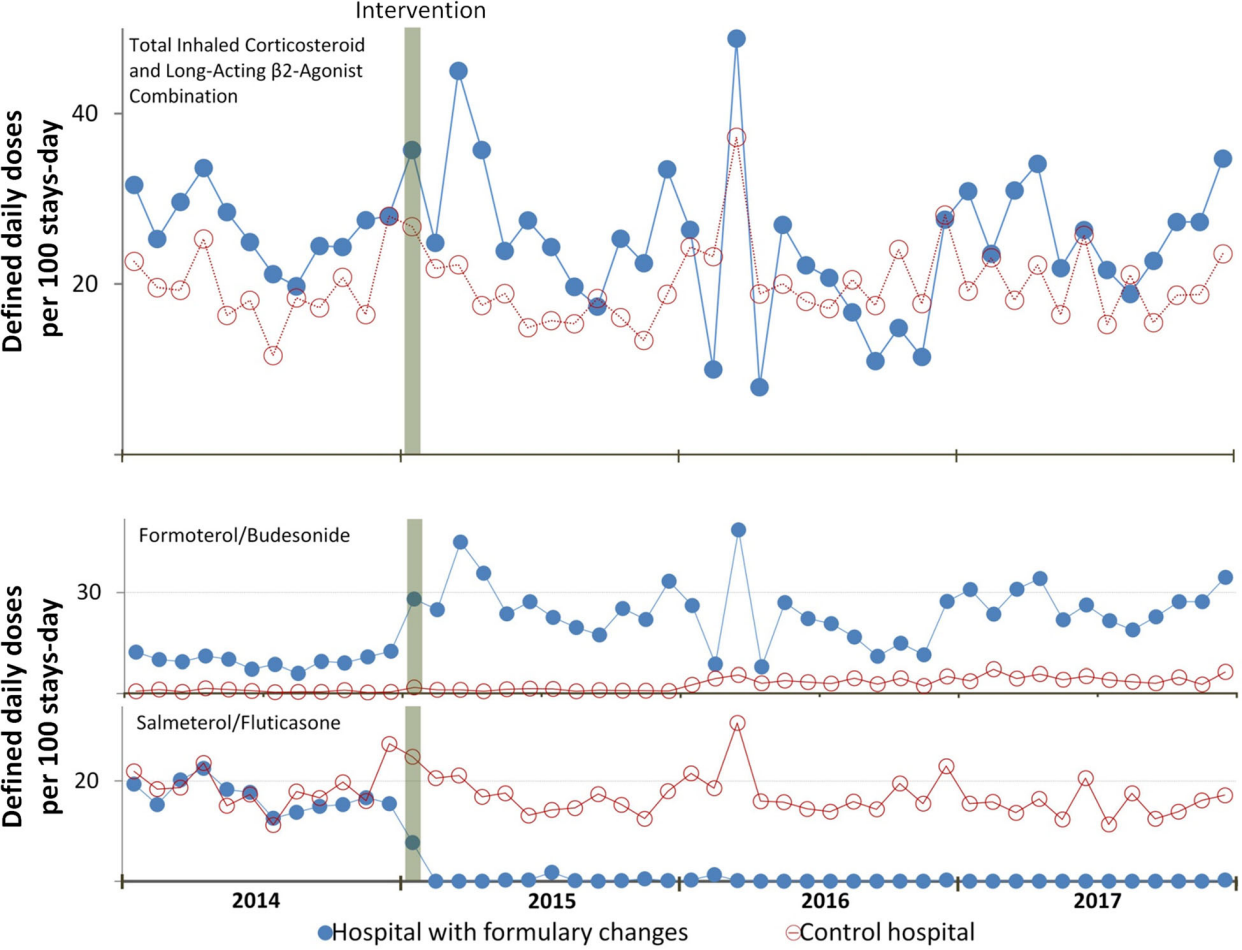
Trends in hospital total inhaled corticosteroid and long-acting beta2-agonist combination utilization

Table 2 shows the results of the ITS. Figure 1 depicts the monthly trend in DDD/100 b-d for the sum of the two ICS/LABA in the formulary and for each individually. There were significant differences between the intervention and control groups.

**Table 2:** Interrupted time series segmented regression analysis with control group of inhaled corticosteroid and long-acting β2-agonist combinations (ICS-LABA)

	Pre-intervention trend		Post-intervention			
			Immediate impact of the formulary change		Change in trend after the formulary change	
	Coefficient	95% confidence interval	Coefficient	95% confidence interval	Coefficient	95% confidence interval
**DDD/100 stays-day**						
Total ICS-LABA	− 0.464	− 2.117 to 1.188	3.344	− 10.312 to 17.002	0.317	− 1.359 to 1993
Formoterol/budesonide^a^	0.012	− 1.438 to 1.464	15.830*	3835 to 27,825	− 0.200	− 1.672 to 1,272
Salmeterol/fluticasone^b^	− 1.043	− 2.483 to 0.396	− 7.792*	− 15.113 to − 0.471	1.075	− 0.37 to 2.521
**Inpatient expenditure per DDD**						
Total ICS-LABA	− 0.003	− 0.119 to − 0.113	− 0.953*	− 1.733 to − 0.174	0.009	− 0.108 to 0.127
**DDD/TID**						
Total ICS-LABA	− 0.405	− 1.046 to 0.235	0.994	− 1.484 to 3.474	0.348	− 0.290 to 0.986
Formoterol/budesonide	0.066	− 0.102 to 0.235	− 0.015	− 0.820 to 0.790	− 0.068	− 0.238 to 0.101
Salmeterol/fluticasone	− 0.039	− 0.194 to 0.115	− 0.143	− 0.991 to 0.704	0.034	− 0.121 to 0.190
Formoterol/beclometasone	− 0.008	− 0.141 to 0.123	− 0.182	− 0.817 to 0.453	− 0.030	− 0.164 to 0.102
Formoterol/fluticasone	0.006	− 0.021 to 0.033	0.107	− 0.103 to 0.319	0.001	− 0.026 to 0.029
Vilanterol/fluticasone	0.008	− 0.01 to 0.027	− 0.023	− 0.154 to 0.107	− 0.017	− 0.037 to 0.002
**Outpatient expenditure per DDD**						
Total ICS-LABA	− 0.405	− 1.046 to 0.235	0.994	− 1.484 to 3.474	0.348	− 0.290 to 0.986

^a^The only ICS-LABA that remained in the intervention hospital drug formulary after the intervention

^b^After the intervention, this ICS-LABA was removed from the intervention hospital drug formulary (not at the control hospital)

*p < 0.05

The results were as follows:

(1) In the case of the formoterol/budesonide combination medication which was retained in the formulary, there was an immediate increase in intra-hospital use of 173.2% (95% CI 47.3–299.0%), and

(2) In the case of the salmeterol/fluticasone combination medication which was withdrawn from the formulary, there was an immediate reduction of 94.4% (95% CI 77.9–111.9%) in intra-hospital use.

### Out-of-hospital use

**Fig. 2:**
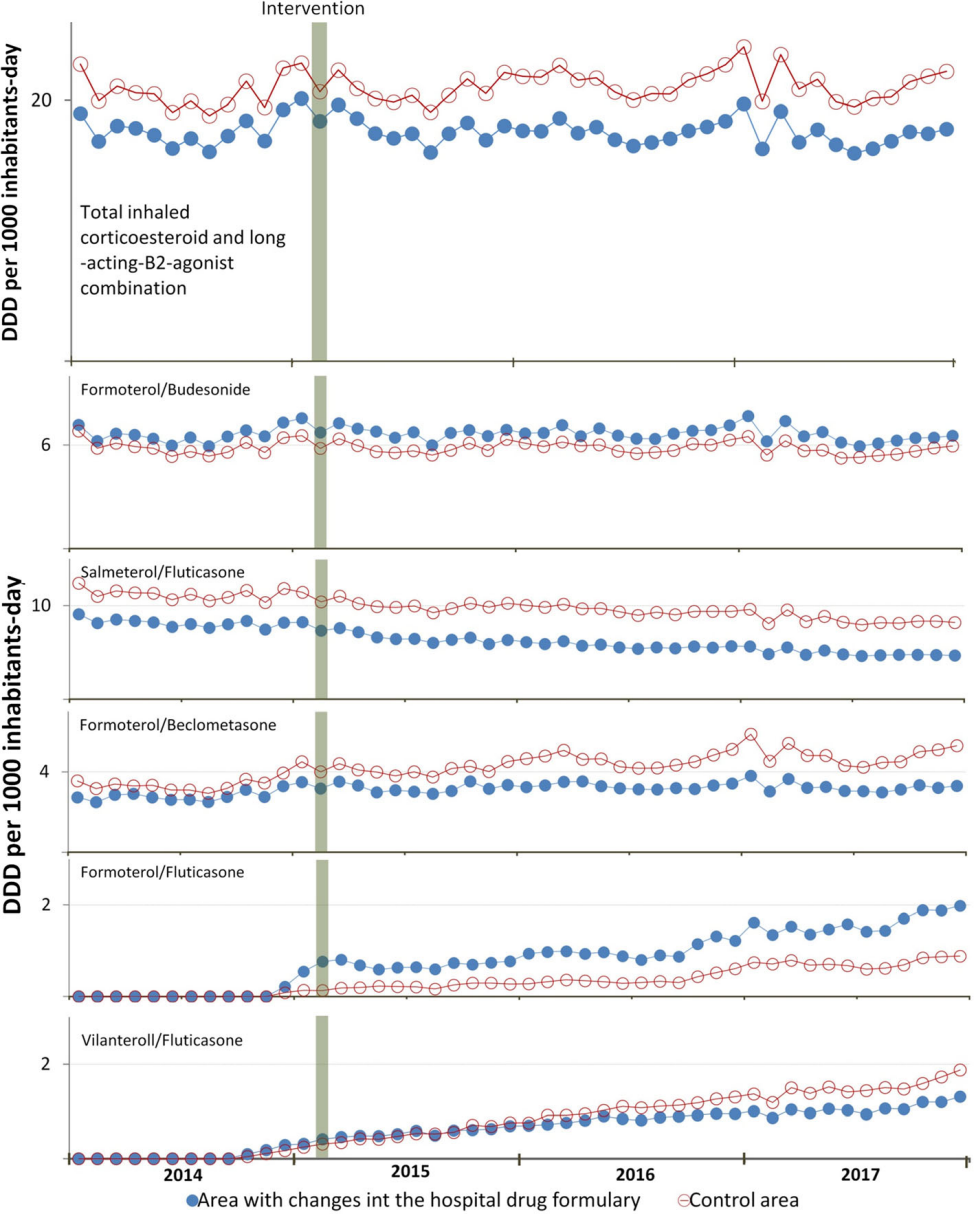
Trends in out-of-hospital total inhaled corticosteroid and long-acting beta2-agonist combination utilization

Table 2 shows the results of the segmented regression. Figure 2 depicts the monthly trend in out-of-hospital DDD/TID for the sum of the five ICS/LABA and for each individually.

In the case, both of the ICS/LABA which was withdrawn (salmeterol/fluticasone) and that which was retained (formoterol/budesonide) in the formulary, there were no significant changes at an out-of-hospital level when compared to the control group.

Similarly, there were no significant changes in the remaining drug presentations in the same group (formoterol/beclometasone, vilanterol/fluticasone, formoterol/fluticasone), when the model was adjusted for the control group.

Likewise, the use of other pulmonary inhalation therapy drugs with indication for asthma or COPD showed no significant shifts following the change in the formulary.

### Costs

**Fig. 3:**
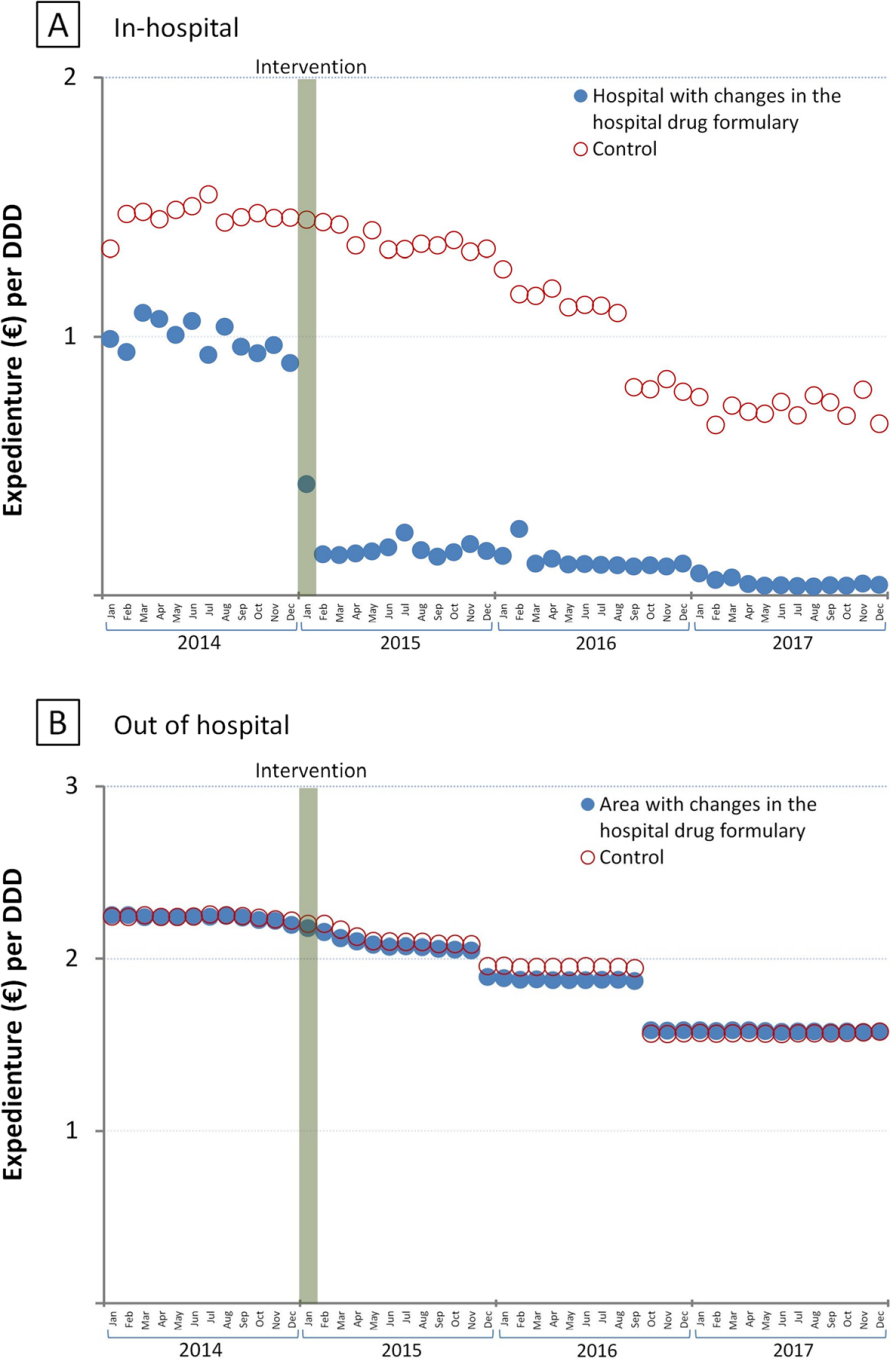
Trends in expenditure (€) per DDD for total inhaled corticosteroid and long-acting beta2-agonist combination utilization

Figure 3 shows that the intra-hospital cost per DDD of total ICS/LABA underwent a significant immediate post-intervention reduction of 75.9% (95% CI 82.8–68.9%) (Table 2), which entailed a savings of €27,564 over 35 months.

At the intervention hospital, the cost/DDD was observed to fall until reaching practically zero, but this did not occur at the control hospital.

At an out-of-hospital level, costs/DDD were practically the same in the two groups throughout the study period (Table 2 and Fig. 3).

No significant differences were found between the intervention and control areas in respiratory system medications other than ICS/LABA.

## Sensitivity analysis

To evaluate the role of possible time-dependent confounding factors in the results, a traditional ITS analysis without a control group was performed (Table S1). It was observed that if no adjustment was made for the control group, the effects of the intervention would indeed prove statistically significant in the out-of-hospital setting, with the use of the combination retained in the formulary as the sole ICS/LABA increasing both immediately (*p* < 0.005) and in the long term (*p* < 0.005).

## Discussion

The results of this quasi-experimental study with control group indicate that a change in the HDF has an important intra-hospital impact, in great measure increasing the prescription of the drug retained and decreasing that of the drug withdrawn from the formulary. As the medication retained was a lower-priced me-too drug, the intervention amounted to an immediate reduction of more than 75% in the cost/DDD of all medications in the class. These results are highly relevant, since they indicate that a simple change in the HDF can yield substantial savings.

Despite the fact that there are HDFs in practically all countries around the world, few studies have evaluated their impact on intra- and out-of-hospital drug prescriptions [14–17] (see Table 3). The important intra-hospital effect found by our study (almost 100% adherence to the HDF, with an immediate increase in the use of the medication retained and a very substantial reduction in the use of the medication withdrawn) is comparable to the findings of the other two studies conducted in the same setting with similar methodology on proton pump inhibitors and on LMWH, as well as another five conducted in different settings on the influence of hospital physicians’ prescribing on that of primary care physicians [2, 5, 14, 15, 29, 30].

**Table 3:** Comparison with other studies which evaluate the out-of-hospital impact of changes in a hospital drug formulary

Author/year	Country	Setting	Therapeutic group	Methodology	Pre-/post-intervention period	Other contextual factors	Effect
Vázquez-Mourelle et al./2017 [16]	Spain	Public district hospital	PPIs	Interrupted time series analysis	1 January 2013/31 December 2015 (36 months) Inclusion of omeprazole	None	Immediate reduction in pantoprazole in hospital outpatient clinics and long-term reduction in primary care. Sharp and long-term rise in the percentage of omeprazole over total PPIs in hospital out-patient units and primary care respectively.
Vázquez-Mourelle et al./2019 [17]	Spain	Public tertiary university teaching hospital	LMWH	Interrupted time series analysis with control group	1 January 2011/31 December 2016 (72 months) Withdrawal of bemiparin and dalteparin Restriction on use of tinzaparin Maintenance of enoxaparin	None	Immediate significant reduction of 55.6% in the medication that was withdrawn and a 9.0% reduction in the medication that was restricted. Immediate 32.6% increase in the drug retained as freely prescribable.
Gallini et al./2013 [14]	France	National health system	Serotonin antagonists LMWH EPO PPIs ACE inhibitors ARBs Statins AAAs SSRIs	Ecological study of spatial clusters, analysed by multivariate linear regression with instrumental variables.	NA (cross sectional)	None	Positive influence of hospital-prescribed medications on community prescriptions. This influence varied, both with drug class, proving stronger in the classes used for the cardiovascular system, and with hospital size.
Larsen et al./2014 [15]	Denmark	Public tertiary university teaching hospital	PPIs (policy of replacing an expensive PPI (esomeprazole) with less expensive PPIs)	Pre-post, descriptive statistics in exact numbers and percentages	1 January 2009/31 December 2010 (policy change: switch to using a less expensive PPI)	Esomeprazole was offered to hospital with a 90% discount, which was an expensive PPI out-of-hospital.	The probability of having an expensive PPI after hospitalisation decreased from 13.4 to 6.5%, while use of recommended PPIs increased.

*LMWH* low molecular weight heparins, *EPO* erythropoietin, *PPIs* proton pump inhibitors, *ACE inhibitors* angiotensin-converting enzyme antagonists inhibitors, *ARBs* angiotensin-receptor blockers, *AAAs* alpha-adrenoreceptor antagonists, *SSRIs* selective serotonin re-uptake inhibitors

The possible impact at an out-of-hospital level is a relevant aspect for ascertaining the induction effect of the hospital intervention on primary care. The absence of effect on out-of-hospital drug prescriptions is in contrast to the results reported by two studies undertaken by our group on proton pump inhibitors and on LMWH, and other studies in other settings [2, 5, 14, 15]. This contrast in results with respect to the studies conducted by our group [16, 17] cannot be attributed to the health-care setting (since all addressed the intervention at the same hospital under the same health service). The main contextual factors which could account for this absence of effect are the following (see Table 3):

(1) Firstly, the therapeutic group studied (bronchodilators versus PPI or LMWH). According to Gallini et al., the therapeutic group does influence the impact of hospital prescriptions on out-of-hospital prescriptions, with the classes of drug used for the cardiovascular system (e.g. LMWH) being the most affected, and

(2) Secondly, the appearance of two new drugs (formoterol/fluticasone and vilanterol/fluticasone) in the community setting for the same indications, overlapping in time with the intervention. Despite the fact that these new, recently marketed drugs were not included in any HDF, they swiftly captured the out-of-hospital market—as is often the case with this class of drugs —[4] in detriment to traditional me-too drug use. Some studies claim that the speed of this shift is attributable, among other factors, to the considerable influence exerted by the pharmaceutical industry’s strong promotional campaign that accompanies any new-product launch [31–34]. Lastly, one cannot rule out the possibility that there may be more studies in which changes in the HDF have not led to an out-of-hospital effect, but which have not been reported (publication bias) due to the absence of the expected and/or noteworthy results.

We regard the matter of costs as very important, since changes in the HDF come at almost zero cost and instantaneously result in almost 100% adherence, with a reduction in costs of over €27,000 across the post-intervention months studied. Hence, even though the out-of-hospital effects may be small and non-significant, the intra-hospital impact could in itself justify the intervention.

### Advantages and limitations

One of the strengths of our study lies in its methodology: the ITS design enables control by design of potentially confounding variables that remain unchanged across the study period (socio-demographic variables, determinants of prescribing, and determinants of drug supply), since these remain constant or with minimal variations across the pre- and post-intervention periods [20, 21].

Another strength is the use of ITS with a control group, especially in view of the fact that most ITS studies do not generally use one [22]. The advantage of this methodology is that it allows one to control for the effect of external factors which occur at the same time as the intervention and might otherwise confound the action or the treatment. This aspect is highly relevant, since in the present case, without such adjustment (see Table S2), the conclusions obtained at an out-of-hospital level would be different (i.e. without a control group significant changes are indeed in evidence). This indicates that there seems to be time-dependent confounding which could not have been controlled for without the use of a control group.

One limitation is the short temporal window prior to the intervention (13 measurements), since a key aspect of ITS analyses is the assumption of trends preceding the intervention in order to extrapolate predictions that subsequently occur [26, 27]. In our case, however, we consider these sufficient to capture the preceding trend, which is practically non-existent in all the dependent variables (see Table 2). Hence, in our study, the principal effect of the short time window prior to the intervention would be to reduce statistical power. Even so, our sensitivity analysis without a control group shows that there is sufficient power to detect statistically significant effects (see Table S2).

Moreover, as with all ecological studies, every relationship of causality must be interpreted with caution. When it comes to extrapolating the results, one has to bear in mind the fact that the Spanish health system finances practically all me-too drugs at an out-of-hospital level, and this is not necessarily the case elsewhere, e.g. in countries such as the USA or New Zealand [35–37].

## Conclusions and implications

The change in the hospital drug formulary proved to be cost-effective in terms of intra-hospital prescription of the ICS/LABA therapeutic group. These effects were immediate, with a high level of treatment adherence. Yet, the results of this study indicate that these changes in hospital prescribing patterns did not generate induction in prescribing in the out-of-hospital setting, which indicates in turn that the effect of induction cannot be generalised to all classes of medications and situations. Hence, there is a need for more studies that would make it possible to identify which factors are associated with out-of-hospital impact. At all events, changes in the HDF are very effective, though their effect may well be restricted to the hospital setting.

## Supplementary information

**Additional file 1: Table S1.** Interrupted time-series segmented regression analysis without control group of inhaled corticosteroid and long-acting β2-agonist combinations (ICS-LABA).

**Additional file 2: Table S2.** Number of measurements, minimum, maximum, mean and standard deviation pre- and post-intervention in the intervention and control group.

## Data Availability

The datasets used and/or analysed during the current study are available from the corresponding author on reasonable request.

## Abbreviations

ICS/LABA: Inhaled corticosteroids and long-acting beta2-agonists
DDD: Defined daily doses
DDD/TID: Defined daily doses per 1000 inhabitants per day
DDD/100 b-d: Defined daily doses per 100 bed-days
