# Supplementary material for: Impact of a change of bronchodilator medications in a hospital drug formulary on intra- and out-of-hospital drug prescriptions: interrupted time series design with comparison group
Source: Implement Sci. 2020 May 14;15:33. doi: 10.1186/s13012-020-00996-y (PMC7227340; doi:10.1186/s13012-020-00996-y)
Supplement: Supplementary file 1 — Additional file 1: Table S1. Interrupted time-series segmented regression analysis without control group of inhaled corticosteroid and long-acting β2-agonist combinations (ICS-LABA). [file 13012_2020_996_MOESM1_ESM.doc]

| Table S1. Interrupted time-series segmented regression analysis without control group of inhaled corticosteroid and long-acting β2-agonist combinations (ICS-LABA). | | | | | | | |
| --- | --- | --- | --- | --- | --- | --- | --- |
|  |  | Pre-intervention  trend | | Post-intervention | | | |
|  |  |  |  | Immediate impact of the formulary change | | Change in trend after the formulary change | |
|  | | Coefficient | 95% Confidence Interval | Coefficient | 95% Confidence Interval | Coefficient | 95% Confidence Interval |
| **DDD/ 100 stays-day** | | | | | | | |
| Total ICS-LABA | | -0.464 | -1.950 to 1.021 | 3.452 | -8.859 to 15.765 | 0.351 | -1.157 to 1.860 |
| Formoterol/Budesonide^a^ | | -0.038 | -1.494 to 1.417 | 16.446* | 4.384 to 28.508 | -0.033 | -1.511 to 1.445 |
| Salmeterol/Fluticasone^b^ | | -0.425* | -0.688 to -0.163 | -12.993* | -15.172 to -10.815 | 0.384* | 0.117 to 0.651 |
| **Inpatient expenditure**  **per DDD** | | | | | | | |
| Total ICS-LABA | | -0.008* | -0.016 to -0.000 | -0.703* | -0.768 to -0.638 | 0.002 | -0.005 to 0.010 |
| **DDD/TID** | | | | | | | |
| Total ICS-LABA | | -0.0525* | -0.082 to -0.022 | 0.8849 | -0.117 to 1.887 | 0.0433* | 0.001 to 0.084 |
| Formoterol/Budesonide | | -0.035* | -0.051 to -0.019 | 0.756* | 0.331 to 1.181 | 0.030* | 0.010 to 0.049 |
| Salmeterol/Fluticasone | | -0.065* | -0.086 to -0,044 | -0.244 | -0.631 to 0.142 | 0.013 | -0.003 to 0.030 |
| Formoterol/Beclometasone | | 0.0510* | 0,033 to 0.068 | 0.467* | 0.218 to 0.715 | -0.042* | -0.058 to -0.026 |
| Formoterol/Fluticasone | | 0.000 | -0.001 to 0.003 | 0.216* | 0.076 to 0.355 | 0.025* | 0.007 to 0.042 |
| Vilanterol/Fluticasone | | 0.001* | 0.000 to 0.002 | 0.140* | 0.051 to 0.229 | 0.007* | 0.002 to 0.013 |
| **Outpatient expenditure**  **per DDD** | | | | | | | |
| Total ICS-LABA | | -0.000 | -0.001 to 0.000 | -0.030 | -0.060 to -0.000 | -0.004* | -0.008 to -0.001 |
| a) The only ICS-LABA that remained in the intervention hospital drug formulary after the intervention.  b) After the intervention, this ICS-LABA was removed from the intervention hospital drug formulary (not at the control hospital).  * *p* < 0.05. | | | | | | | |
