# Supplementary material for: Impact of a change of bronchodilator medications in a hospital drug formulary on intra- and out-of-hospital drug prescriptions: interrupted time series design with comparison group
Source: Implement Sci. 2020 May 14;15:33. doi: 10.1186/s13012-020-00996-y (PMC7227340; doi:10.1186/s13012-020-00996-y)
Supplement: Supplementary file 2 — Additional file 2: Table S2. Number of measurements, minimum, maximum, mean and standard deviation pre- and post-intervention in the intervention and control group. [file 13012_2020_996_MOESM2_ESM.docx]

|  | **Intervention** | | **Control** | |
| --- | --- | --- | --- | --- |
|  | **Pre** | **Post** | **Pre** | **Post** |
| **Observations**  **Missing data** | 13  0 | 35  0 | 13  0 | 35  0 |
| **Intra-hospital**  DDD/100 b-d for ICS/LABA  Cost (€/DDD) | **min max mean SD**  19.76 35.76 27.28 4.64  0.43 1.09 0.95 0.17 | **min max mean SD**  7.93 48.81 24.71 8.89    0.04 0.26 0.12 0.08 | **min max mean SD**  11.62 27.97 20.04 4.61  1.34 1.55 1.46 0.05 | **min max mean SD**  13.41 37.26 19.94 4.54    0.66 1.45 1.05 0.29 |
| **Out-of-hospital**  DDD/TID for ICS/LABA  Cost (€/DDD) | **min max mean SD**  16.08 20.16 17.75 1.21    2.17 2.25 2.23 0.02 | **min max mean SD**  15.95 19.74 17.57 0.93    1.57 2.15 1.8 0.22 | **min max mean SD**  18.82 22.86 20.67 1.37  2.20 2.26 2.24 0.01 | **min max mean SD**  19.10 24.11 21.17 1.15    1.56 2.20 1.85 0.25 |

Table S2. Number of measurements, minimum, maximum, mean and standard deviation pre- and post-intervention in the intervention and control group.
